# Cystic gastric metastasis from pancreatic cancer

**DOI:** 10.1186/s40792-018-0443-2

**Published:** 2018-04-10

**Authors:** Naoki Umezaki, Daisuke Hashimoto, Shigeki Nakagawa, Takanobu Yamao, Masayo Tsukamoto, Yuki Kitano, Kota Arima, Kensuke Yamamura, Tatsunori Miyata, Hirohisa Okabe, Akira Chikamoto, Fujio Matsumura, Hideo Baba

**Affiliations:** 1 Department of Gastroenterological Surgery, Kumamoto University Graduate School of Medical Sciences, 1-1-1 Honjo, Kumamoto, 860-8556 Japan; 2 Department of Gastroenterological Surgery, Omuta Tenryo Hospital, 1-100 Tenryo,, Omuta, 836-8566 Japan

**Keywords:** Pancreatic cancer, Metastasis, Submucosal tumor, Operation, Chemotherapy

## Abstract

Gastrointestinal tract metastasis from pancreatic cancer is quite rare. We present the case of a 58-year-old male patient who underwent distal pancreatectomy for pancreatic body cancer 5 years prior. Four years after the initial operation, a 15-mm cystic submucosal tumor was found in the antrum of the stomach. Because the tumor had grown to 25 mm and the level of carcinoembryonic antigen in the cystic fluid derived by ultrasound-guided fine-needle aspiration biopsy was high, partial resection of the stomach was performed 5 years after the distal pancreatectomy. Pathological diagnosis was gastric metastasis of pancreatic cancer. The patient has been alive without recurrence for 13 months after the resection of the cystic tumor. We are not aware of any similar cases of cystic gastric metastasis from pancreatic cancer published in the English literature.

## Background

Pancreatic cancer is the most lethal of the common human malignancies and the fifth leading cause of cancer-related death in Japan [1]. Even after pathologically curative resection, most patients have disease recurrence [2]. Multimodality therapy, such as the combination of surgery and chemotherapy, has been important for pancreatic cancer [2].

Pancreatic cancer often develops both hematogenous and lymphatic metastases, with metastasis usually occurring in the liver, lung, peritoneum, or bones [3]. Gastric involvement is occasionally observed in pancreatic cancer patients, although it results not from metastasis but from direct invasion [4].

Gastrointestinal metastasis from pancreatic cancer is quite rare [5]. In addition, metastatic tumors from pancreatic cancer are usually solid tumors. As we are not aware of a similar case in the English literature, we would like to present a case of cystic gastric metastasis from pancreatic cancer.

## Case presentation

**Fig. 1:**
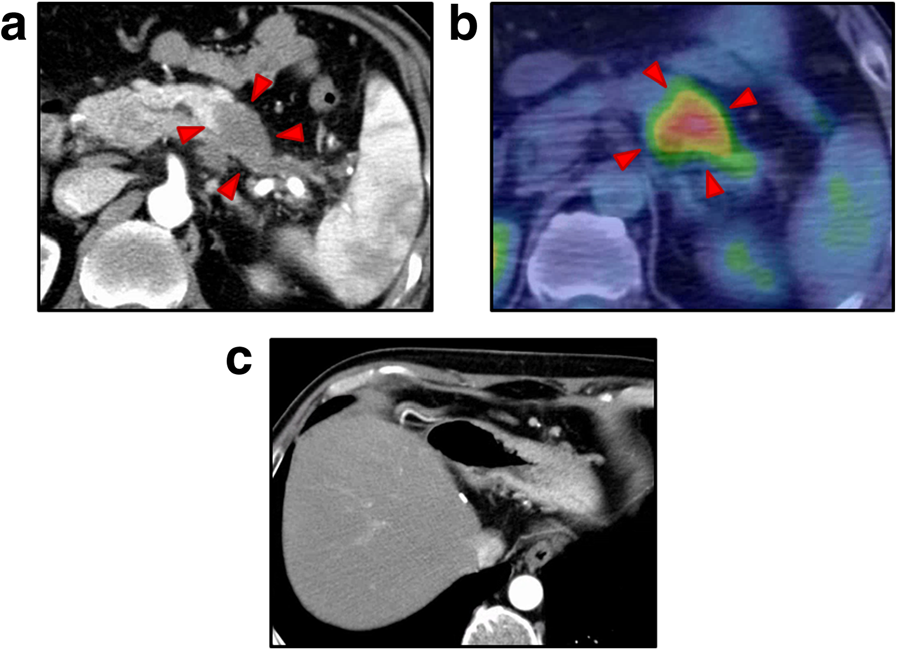
Preoperative imaging of the primary pancreatic cancer. Enhanced computed tomography (CT) showed a 30-mm solid tumor in the tail of the pancreas (**a**, arrowheads). Positron emission tomography–CT showed abnormal accumulation in the tumor (**b**, arrowheads). There was no gastric before the initial surgery (**c**)

A 58-year-old male patient was found to have hyperamylasemia upon medical examination. A contrast-enhanced computed tomography (CT) scan revealed a 30-mm low-density solid tumor in the tail of the pancreas (Fig. 1a). Positron emission tomography–computed tomography (PET-CT) showed a maximum standardized uptake value (SUV max) of the tumor of 3.5 to 3.7 (Fig. 1b). There was no distant metastasis on enhanced CT or PET-CT. The serum levels of carcinoembryonic antigen (CEA) and carbohydrate antigen 19-9 (CA 19-9) were not elevated (CEA = 1.0 ng/mL, CA 19-9 = 5.8 U/ml). We diagnosed a resectable pancreatic tail cancer. Moreover, prior to the initial surgery, there was no tumor lesion in the gastric antrum (Fig. 1c), and endoscopic ultrasonography (EUS) was not performed.

**Fig. 2:**
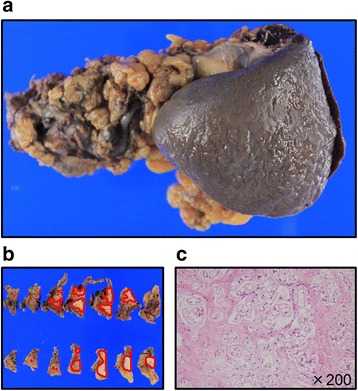
Pathological findings of the primary pancreatic cancer. Macroscopically, the size of the primary pancreatic cancer was 3.9 × 2.6 cm (**a**, **b**). Microscopically, pathological diagnosis was moderately > well-differentiated tubular adenocarcinoma (**c**)

The patient underwent distal pancreatectomy with D2 lymph node dissection and splenectomy (Fig. 2a,b). The intraoperative peritoneal lavage cytology was negative. Pathological diagnosis was tubular adenocarcinoma (moderately > well-differentiated), Pbt, TS2, tumor diameter of 39 × 26 mm, nodular type, pT3, int, INFβ, ly0, v1, ne2, mpd0, pCH0, pDU0, pS1, pRP1, pPVsp1, pAsp1, pPL0, pOO0, pPCM0, pBCMX, pDPM0, pN0, M0, CY0, pStage IIA, D2, and pR0 according to the 7th edition of Japanese Pancreatic Society staging system and T3N0M0 Stage IIA (pathological grade G2) in the 7th edition of the AJCC/UICC staging system (Fig. 2c). The patient received adjuvant chemotherapy with 16 courses of gemcitabine.

**Fig. 3:**
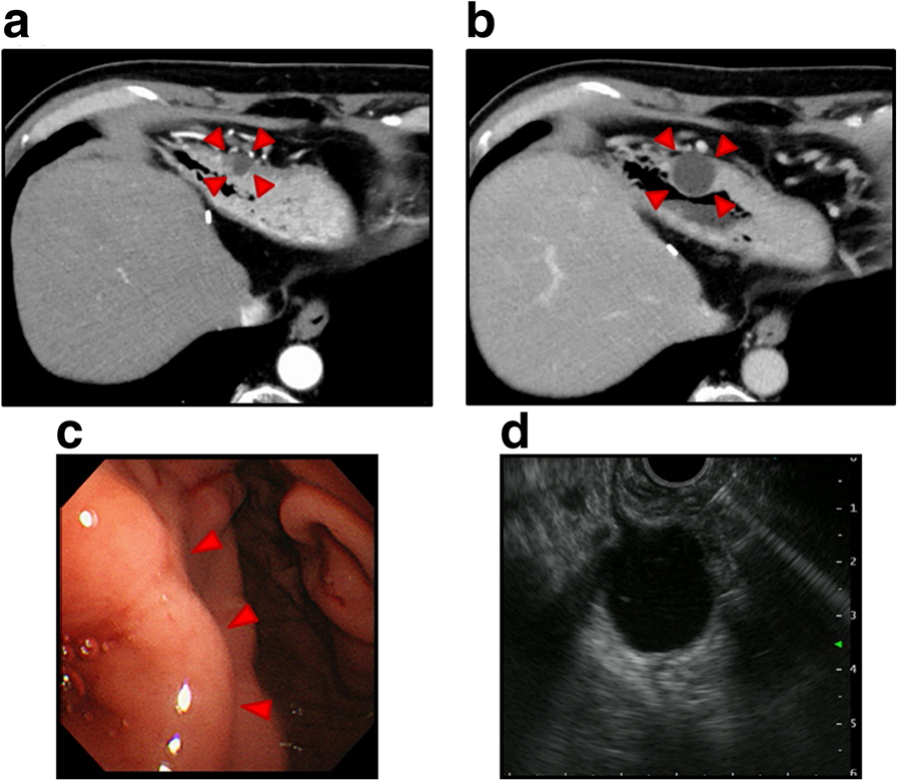
Preoperative imaging of the cystic tumor in the gastric antrum. Enhanced CT showed a 15-mm cystic lesion of the gastric antrum (**a**, arrowheads) 4 years after the initial operation. Six months later, the cystic lesion had grown up to 25 mm (**b**, arrowheads). Endoscopy revealed no epithelial lesion (**c**, arrowheads). Endoscopic ultrasonography showed a submucosal tumor (**d**)

**Fig. 4:**
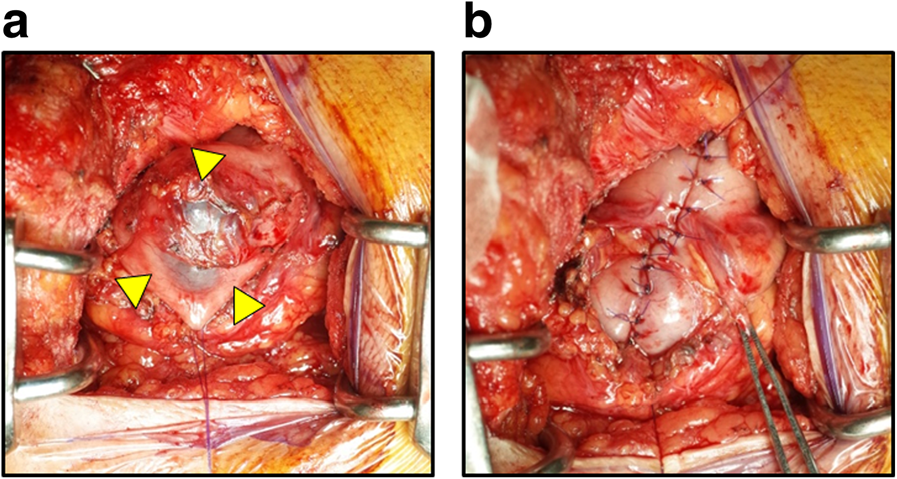
Operative findings of the cystic tumor in the gastric antrum. Laparotomy showed the cystic tumor at the anterior wall of the stomach (**a**). Partial gastrectomy was performed, and the defect of the stomach was closed (**b**)

Abdominal enhanced CT showed a 15-mm cystic tumor in the gastric antrum 4 years after the surgery (Fig. 3a). Tumor markers were not elevated. Because the cystic lesion had grown to 25 mm, 6 months later (Fig. 3b), upper gastrointestinal endoscopy and EUS were performed. Endoscopy revealed no epithelial lesion in the antrum (Fig. 3c); however, EUS showed a submucosal tumor (Fig. 3d). EUS-guided fine-needle aspiration biopsy (EUS-FNA) was performed, and light brown serous cystic fluid was collected. Cytological diagnosis of the cyst fluid was class III. Interestingly, CEA of the cyst fluid was 6770 ng/mL, whereas serum CEA and CA19-9 levels were 1.5 ng/mL and 6.6 U/mL, respectively. Thus, the cystic tumor could have been gastric metastasis from pancreatic cancer. The tumor existed at the anterior wall of the stomach; so, it was not considered as direct invasion from the pancreatic cancer. Because there was no other metastasis or recurrence, we planned resection of the cystic tumor. After laparotomy and adhesiolysis, the cystic tumor was detected at the anterior wall of the stomach (Fig. 4a). Partial gastrectomy was performed, and the defect of the stomach was closed in the minor axis direction (Fig. 4b). The intraoperative peritoneal lavage cytology result was negative. There were no postoperative complications, and the postoperative course was good.

**Fig. 5:**
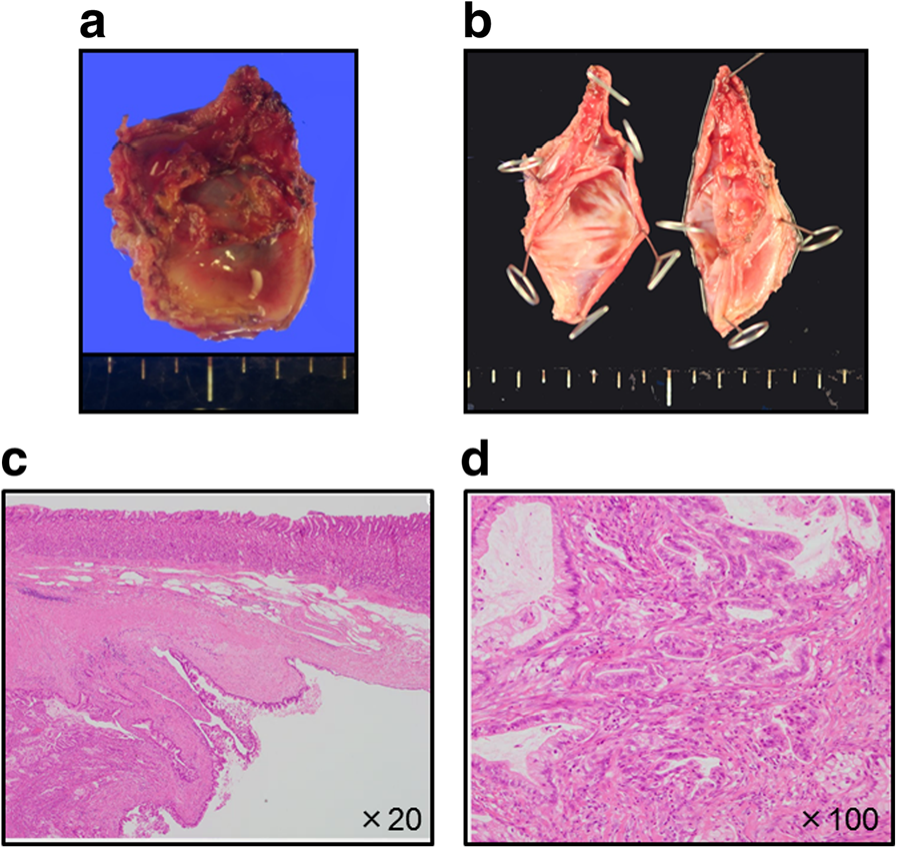
Pathological findings of the cystic gastric metastasis. Macroscopically, there was a 25-mm round cystic tumor (**a**, **b**). Microscopically, the cystic tumor existed in the submucosal layer (**c**). The tumor comprised tubular adenocarcinoma cells (**d**). Invasion of the adenocarcinoma cells did not reach the epithelium

Macroscopically, there was a 25-mm round, cystic tumor (Fig. 5a,b). Microscopically, adenocarcinoma arising from the mucinous atypical acinar epithelium was observed from the intrinsic muscle layer of the stomach wall to the subserosal tissue (Fig. 5c). Adenocarcinoma mainly took on a cystic structure, in contrast to primary pancreatic cancer, which presented as a solid adenocarcinoma tumor with rich stromal cells. Some parts of the tumor represented a papillary structure with irregular ducts (Fig. 5d). There was no tumor proliferation in the epithelium of the gastric mucosa. Thus, the pathological diagnosis was adenocarcinoma, consistent with metastasis of pancreatic cancer.

The patient received adjuvant chemotherapy with S-1. The patient has survived without obvious recurrence or metastasis for 13 months after the partial gastrectomy.

Generally, metastasis or recurrence of pancreatic cancer is found in the liver, lymph nodes, peritoneum, and lung [4, 6]. Involvement of the stomach due to direct invasion by cancer of the pancreatic body and tail has occasionally been observed [4], but gastric metastasis from pancreatic cancer is quite a rare event. Possible mechanisms of cancer involvement of the stomach are direct invasion, intraoperative seeding, hematogenous metastasis, lymphatic metastasis, and intraluminal or intramural dissemination [7].

In our case, a growing cystic tumor in the gastric antrum wall was detected. Because the tumor was located in the submucosal layer and separated from the space where the primary pancreatic cancer existed or the pancreatic cut ended, we diagnosed it as hematogenous metastasis to the stomach.

Sasajima et al. indicated that it was difficult to distinguish between gastric metastasis and direct invasion of the stomach in advanced cancer [5]. Autopsy studies often lead to the diagnosis of metastasis when cancer cells are detected histologically in other organs [8]. Gastric metastasis from pancreatic cancer was described as a rare condition in those studies [4, 6]. Campoli et al. reported 20 patients with metastatic cancer of the stomach and indicated that the primary sites were the esophagus, skin, lung, cervix, breast, sigmoid colon, and testis, but not the pancreas [9]. Oda et al. reported that 347 cases of gastric metastases from solid malignant tumors were identified from a series of 6380 autopsy reports; thus, the incidence of gastric metastasis was 5.4% [10]. In their study, the most common primary malignancy of gastric metastasis was lung cancer, followed by breast and esophageal cancers [10]. Additionally, they performed autopsies on 209 patients with pancreatic cancer and reported only 2 cases with gastric metastasis [10]. These results indicate that the pancreas is a rare primary lesion of gastric metastasis.

It is also difficult to distinguish a gastric metastasis of pancreatic cancer from a heterochrony primary pancreatic cancer originated from aberrant pancreas in the stomach. Gillou et al. described that the possibility of a tumor from ectopic pancreatic tissue origin is acceptable only if the following three conditions are met: (1) the tumor must be found within or close to the ectopic pancreatic tissue, (2) direct transition between pancreatic structures and the carcinoma must be observed, and (3) the nonneoplastic pancreatic tissue must comprise, at least, fully developed acini and ductal structures [11]. Because all of the above conditions are not found in the tumor microscopically in our case, we thought the tumor was not a primary pancreatic cancer originated from ectopic pancreas in the stomach.

In addition, metastatic tumors from pancreatic cancer, such as liver metastasis and lymph node metastasis, are usually solid tumors. However, our case presented with a cystic metastatic tumor. English language publications were systematically searched to find similar cases in the National Library of Medicine (MEDLINE [PubMed, available at: http://www.pubmed.com/]) published between 1970 and 2017 using the medical terms “pancreatic cancer,” “gastric metastasis,” and “cystic tumor”. However, no similar cases were found. In addition, no cases of cystic metastasis of the stomach in other primary malignancies were found. It is known that a metastatic lung tumor can present as a cystic lesion [12]. The mechanism of how it became a cystic tumor has been considered such as ischemic necrosis of the center of the tumor.

Several papers have reported good prognoses after resection for solitary metastasis from pancreatic cancer, in the lung or remnant pancreas, which developed long after the initial pancreatectomy for the primary lesion [13–15]. Therefore, partial gastrectomy for this case seems to be a feasible operation.

## Conclusions

The gastric metastasis from pancreatic cancer is quite rare. In addition, the reported patients developed a cystic submucosal tumor. In carefully selected patients, we believe that surgical excision of a solitary metastasis like our case may prolong survival time.

## Abbreviations

CA19-9: Carbohydrate antigen 19-9
CEA: Carcinoembryonic antigen
CT: Computed tomography
EUS: Endoscopic ultrasonography
FNA: Fine-needle aspiration biopsy
PET: Positron emission tomography
SUV: Standardized uptake value

## References

[1] Matsuno S, Egawa S, Fukuyama S (2004). Pancreatic cancer registry in Japan: 20 years of experience. Pancreas.

[2] Paulson AS, Tran Cao HS, Tempero MA (2013). Therapeutic advances in pancreatic cancer. Gastroenterology.

[3] Peixoto RD, Speers C, McGahan CE (2015). Prognostic factors and sites of metastasis in unresectable locally advanced pancreatic cancer. Cancer Med.

[4] Cubilla A, Fitzgerald PJ (1978). Pancreas cancer. I. Duct adenocarcinoma. A clinical-pathologic study of 380 patients. Pathol Annu.

[5] Sasajima J, Okamoto K, Taniguchi M (2016). Hematogenous gastric metastasis of pancreatic cancer. Case Rep Gastroenterol.

[6] Blastik M, Plavecz E, Zalatnai A (2011). Pancreatic carcinomas in a 60-year, institute-based autopsy material with special emphasis of metastatic pattern. Pancreas.

[7] Feczko PJ, Collins DD, Mezwa DG (1993). Metastatic disease involving the gastrointestinal tract. Radiol Clin N Am.

[8] Matsuda Y, Hagio M, Naito Z (2012). Clinicopathological features of 30 autopsy cases of pancreatic carcinoma. J Nippon Med Sch.

[9] Campoli PM, Ejima FH, Cardoso DM (2006). Metastatic cancer to the stomach. Gastric Cancer.

[10] Oda I, Kondo H, Yamao T (2001). Metastatic tumors to the stomach; analysis of 54 patients diagnosed at endoscopy and 347 autopsy cases. Endoscopy.

[11] Guillou L, Nordback P, Gerber C (1994). Ductal adenocarcinoma arising in a heterotopic pancreas situated in a hiatal hernia. Arch Pathol Lab Med.

[12] Honda O, Tsubamoto M, Inoue A (2007). Pulmonary cavitary nodules on computed tomography: differentiation of malignancy and benignancy. J Comput Assist Tomogr.

[13] Arnaoutakis GJ, Rangachari D, Laheru DA (2011). Pulmonary resection for isolated pancreatic adenocarcinoma metastasis: an analysis of outcomes and survival. J Gastrointest Surg.

[14] Katz MH, Wang H, Fleming JB (2009). Long-term survival after multidisciplinary management of resected pancreatic adenocarcinoma. Ann Surg Oncol.

[15] Kleeff J, Reiser C, Hinz U (2007). Surgery for recurrent pancreatic ductal adenocarcinoma. Ann Surg.

